# NK Cell Metabolism and Tumor Microenvironment

**DOI:** 10.3389/fimmu.2019.02278

**Published:** 2019-09-24

**Authors:** Iñigo Terrén, Ane Orrantia, Joana Vitallé, Olatz Zenarruzabeitia, Francisco Borrego

**Affiliations:** ^1^Immunopathology Group, Biocruces Bizkaia Health Research Institute, Barakaldo, Spain; ^2^Ikerbasque, Basque Foundation for Science, Bilbao, Spain

**Keywords:** NK cell, metabolism, glucose, glycolysis, amino acid, hypoxia, tumor microenvironment, TME

## Abstract

Natural Killer (NK) cells are characterized by their potential to kill tumor cells by different means without previous sensitization and have, therefore, become a valuable tool in cancer immunotherapy. However, their efficacy against solid tumors is still poor and further studies are required to improve it. One of the major restrictions for NK cell activity is the immunosuppressive tumor microenvironment (TME). There, tumor and other immune cells create the appropriate conditions for tumor proliferation while, among others, preventing NK cell activation. Furthermore, NK cell metabolism is impaired in the TME, presumably due to nutrient and oxygen deprivation, and the higher concentration of tumor-derived metabolic end products, such as lactate. This metabolic restriction of NK cells limits their effector functions, and it could represent a potential target to focus on to improve the efficacy of NK cell-based therapies against solid tumors. In this review, we discuss the potential effect of TME into NK cell metabolism and its influence in NK cell effector functions.

## Introduction

Natural Killer (NK) cells are a promising tool in cancer immunotherapy. Their activation is driven by a balance between activating and inhibitory signals, so they are able to exert antitumor responses without prior sensitization. NK cells have demonstrated their potential in the treatment of several malignancies. However, the efficacy of these cells to treat solid tumors is still unsatisfactory (1–3). One of the main reasons for this limitation is the immunosuppressive effect of the tumor microenvironment (TME). In the TME, several tumor and tumor-associated cells produce and secrete factors that directly or indirectly prevent NK cell activation, including interleukin (IL)-6, IL-10, transforming growth factor-β (TGF-β), prostaglandin E2 (PGE2), and idoleamine 2,3-dioxygenase (IDO) (4, 5). Through these cytokines and factors, tumors are able to downmodulate NK cell activating receptors, such as NKp30, NKp44, or NKG2D (3, 4, 6–8), and tumor necrosis factor-related apoptosis-inducing ligand (TRAIL) (9). Furthermore, in the TME, NK cells receive signals from inhibitory receptors such as CD94/NKG2A, which bind to HLA-E exposed on the surfaces of several solid tumors including lung, pancreas, stomach, colon, head and neck, and liver tumor tissues (10). These immunosuppressive mechanisms mainly alter the balance between activating and inhibitory signals of NK cells, a step that is decisive for NK cell activation. Nonetheless, it should be also considered the effect of the TME in NK cell metabolism, which is essential to display full effector functions (11).

**Figure 1:**
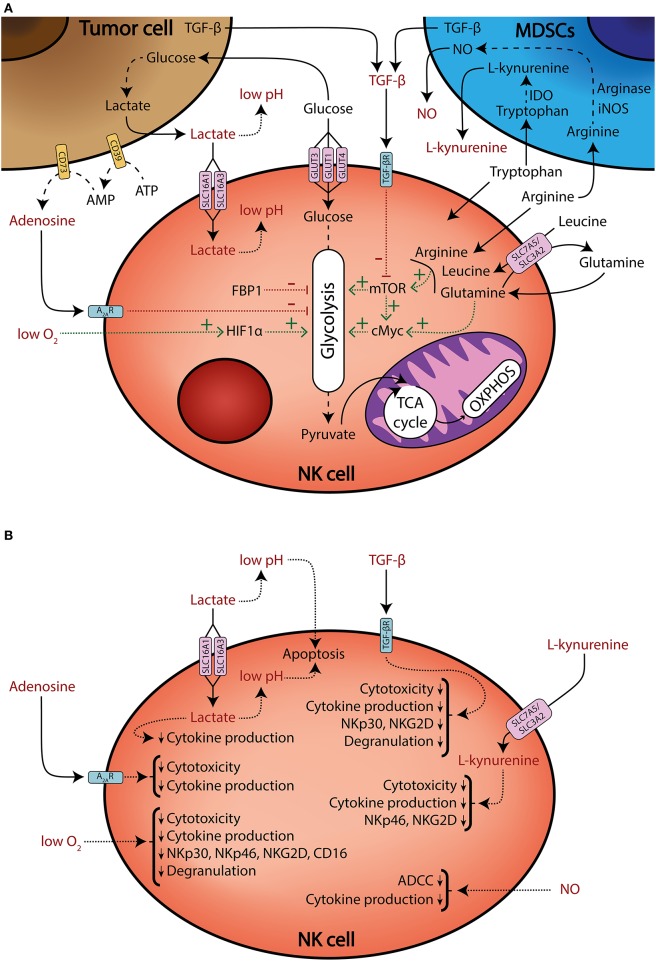
Tumor microenvironment shapes NK cell metabolism and effector functions. **(A)** Schematic representation of multiple factors that modulate NK cell metabolism (black), and factors that modulate metabolism and/or negatively affect effector functions (red). NK cells compete for nutrients against tumor and myeloid-derived suppressor cells (MDSCs). Tumor cells consume large amounts of glucose and produce lactate, which is transported into NK cells through the SLC16A1 and SLC16A3 transporters, impairing ATP production. Tumor cells also generate extracellular adenosine through the ectonucleotidases CD39 and CD73. Extracellular adenosine inhibits NK cell oxidative phosphorylation (OXPHOS) and glycolytic capacity. Additionally, tumor cells increase amino acid consumption and MDSCs upregulate arginase, IDO, and iNOS enzymes, thus generating an amino acid depleted environment and releasing the immunosuppressive metabolites NO and L-kynurenine. Some amino acids and their transport are necessary to sustain mTOR and cMyc signaling, which promote glycolysis. Moreover, mTOR signaling can be also impaired by TGF-β secreted by tumor cells and MDSCs. The glycolytic pathway can be also modulated by the FBP1 enzyme, which is found to be upregulated in the tumor-infiltrating NK cells of some cancers. Finally, high oxygen consumption by tumor cells and disorganized vascularization can generate hypoxic regions. Hypoxia impairs NK cell effector functions, but also sustains HIF1α, which promotes glycolytic metabolism. Solid lines: release or uptake of different nutrients, metabolites, and other factors. Dashed lines: metabolic processing of substrates. Dotted lines: factors that promote or sustain (green lines), or inhibit (red lines), specific pathways. **(B)** A schematic representation of several metabolites and other factors present in the TME that limit NK cell effector functions.

It is now accepted that the metabolic profile of NK cells is different under certain pathologies, such as obesity (12, 13) or viral infection (14). Also, it can be modified by several processes, including education (15, 16), maturation (17), or cytokine stimulation (18–28). The latter is especially relevant because of the potential use of cytokine-stimulated and/or expanded NK cells for adoptive cell therapy in cancer treatment (29–32). In the tumor context, multiple factors converge to modulate NK cell metabolism. For instance, it is known that TGF-β downregulates the expression of activating receptors and effector functions (6, 33–37), but it also limits metabolic changes that accompany cell activation (18, 34). Recently, it has been demonstrated that TGF-β decreases IL-2-induced mitochondrial metabolism, including oxidative phosphorylation (OXPHOS) and maximal respiration in human NK cells (18). Another example of the impact of TME in NK cell metabolism comes from a recent report from patients with colorectal liver metastasis. The authors found that tumor-infiltrating liver-resident NK cells showed signs of mitochondrial stress, including decreased mitochondrial mass and increased reactive oxygen species (ROS) production (38). This reduced mitochondrial metabolism may be an important limitation for NK cell functionality in the TME. In this review, we will discuss how TME could shape NK cell metabolism and thus impair their antitumor activity (Figure 1).

## Tumor-Derived Metabolites

Besides immunosuppressive cytokines, in the TME there is also an accumulation of tumor-derived metabolites, such as adenosine and lactate that limit antitumor responses. Extracellular adenosine concentration is increased in the hypoxic conditions of tumors, and it plays an important role in immune modulation (39). Hypoxia promotes the release of ATP and AMP, and the ectonucleotidases CD39 and CD73 catalyze the conversion of extracellular ATP to AMP, and AMP to adenosine (40). The stimulation of NK cells through the adenosine A_2A_ receptor (A_2A_R), the predominant subtype of adenosine receptor expressed in these cells, has been found to suppress their effector functions (40–43). Following this, it has been reported that adenosine inhibits cytotoxic activity of mouse lymphokine-activated killer (LAK) cells (44). A more recent article has shown that adenosine impairs metabolic activity of IL-12/15-stimulated human NK cells by inhibiting their OXPHOS and glycolytic capacity (45). Interestingly, it was reported that there is an elevated interferon γ (IFNγ) production in the presence of adenosine, along with a decrease in NK cell cytotoxicity, suggesting that the metabolic requirements for these two NK cell effector functions are not the same. Indeed, IL-15-stimulated NK cells showed reduced killing of A549 cancer cells in the presence of adenosine (45). A possible explanation for the elevated IFNγ production may be the downregulation of the *GAPDH* gene observed in IL-12/15-stimulated NK cells exposed to adenosine (45). It has been shown that GAPDH can bind to IFNγ mRNA and prevent its translation (46). However, this transcript-arresting mechanism has not been defined in NK cells yet, and it has to be considered that other mechanisms involved in the regulation of IFNγ production may explain these results.

On the other hand, lactate and low pH have been found to decrease cytotoxic activity of NK cells (47). Exposure of NK cells to lactic acid blocked their IFNγ production following PMA/Ionomycin stimulation (48). A more comprehensive analysis revealed that lactic acid inhibits the upregulation of nuclear factor of activated T cells (NFAT), which is involved in IFNγ transcription (48). Additionally, Brand et al. have also shown that lactic acid uptake by murine NK cells leads to intracellular acidification and to an impaired energy metabolism (measured as intracellular ATP levels) (48). Similar results were obtained in liver-resident NK cells treated with lactic acid, in which intracellular pH and ATP decreased, promoting apoptosis (38). The accumulation of lactate in the TME is mainly due to the metabolic reprogramming of tumors, characterized by primarily using glucose for glycolytic metabolism rather than metabolizing it via OXPHOS. This accelerated glycolysis of cancer cells, induced by multiple factors such as hypoxia and oncogenes (49), may represent a considerable obstacle for NK cell activity, since it is not only causing lactate accumulation but also reducing glucose availability in the TME. Considering that NK cells strongly rely on glucose metabolism to exert their effector functions, as we will discuss in the next section, limiting their key fuel may seriously dampen their antitumor activity. However, not only tumor cells but also many immune cells undergo metabolic reprogramming upon activation, a process that may be especially relevant in the context of the TME and have a significant impact in the tumor progression (50).

## Glucose Restriction

Lymphocytes require glucose to survive and its consumption is increased following activation, to support energetic and biosynthetic demands (51). Glucose can be utilized by NK cells for ATP and NADPH generation through different metabolic pathways, or as a carbon source for other biomolecules such as amino acids and fatty acids (19). It has been reported that NK cells express GLUT1, GLUT3, and GLUT4 (15, 21, 22, 52, 53), three glucose transporters from the GLUT family. Additionally, RNA expression of GLUT8 and H^+^/myo-inositol co-transporter (HMIT or GLUT13) has been also measured in human NK cells (16). However, most studies have been focused on GLUT1, so the expression and regulation of the rest of glucose transporters of the GLUT family are unknown. Upon cytokine-stimulation, NK cells increase GLUT1 expression (21, 22), which is consistent with the augmented glucose uptake and glycolysis that accompanies cell activation (17, 21, 23). Several groups have studied the correlation between the glycolytic pathway and the functionality of activated NK cells, and have shown its relevance in the production of IFNγ and granzyme B, cytotoxicity and proliferative capacity (21, 23–25, 54). These findings are in accordance with those obtained in other lymphocytes. It has been demonstrated that glucose deprivation dampens T cell antitumor activity (46, 55, 56), and that metabolic competition in the TME can regulate cancer progression by impairing antigen-specific responses of tumor-infiltrating T cells (57). Therefore, it is reasonable to hypothesize that in the TME, tumor-driven glucose restriction may reduce glycolysis of NK cells and thus impair their antitumor functions. Cong et al. have addressed this issue by investigating NK cells in a murine model of lung cancer. They have found lower glycolytic rates in NK cells from the lung cancer microenvironment, which also presented attenuated cytotoxicity and cytokine production. Furthermore, Cong et al. have described the increased expression of fructose-1,6-bisphosphatase (FBP1), an enzyme that inhibits glycolysis, in NK cells of the lung cancer microenvironment. More importantly, they have demonstrated that NK cell effector functions can be restored during tumor promotion by inhibiting FBP1 (58). These findings represent a good example of how metabolism can be modulated to improve NK cell antitumor responses.

On another front, Assmann et al. thoroughly analyzed the metabolic reprogramming of cytokine-stimulated NK cells, and described the relevance of citrate-malate shuttle and its regulation by sterol regulatory element-binding proteins (SREBP). They found that SREBP activity is crucial to maintain elevated glycolytic rates and effector functions, including cytotoxicity and IFNγ and granzyme B production (19). Remarkably, some SREBP inhibitors may be increased in the TME, such as 27-hydroxycholesterol, which is found to be elevated in patients with breast, gastric and colorectal cancers (11, 59–62). Considering this fact and that citrate-malate shuttle relies on glucose metabolism, it would be interesting to study the modulation of SREBP in tumor-infiltrating NK cells and to test whether this metabolic pathway configuration is conserved in the TME, where glucose concentration is diminished. Also, it would be of utmost interest to understand the effect of TME in the modulation of other mediators linked to both NK cell metabolism and function, such as AMP-activated protein kinase (AMPK), glycogen synthase kinase 3 β (GSK-3β), diacylglycerol kinases (DGK), regulatory factor X 7 (Rfx7), or inositol-requiring enzyme 1 α (IRE1α) and its substrate X-box-binding protein 1 (XBP1) (63–69). Following this line, it would be also worthwhile to further investigate the role of the mechanistic (or mammalian) target of rapamycin (mTOR), a central metabolic regulator that promotes, among others, the glycolytic pathway. Several authors have pointed the relevance of mTOR for NK cell activation and metabolic reprogramming, and the negative effect of mTOR inhibition in terms of NK cell functionality (12, 17, 21, 23, 26, 70–72). mTOR is sensitive to nutrient availability (73) and can be repressed by TGF-β, which inhibits NK cell metabolism and functionality (18, 33, 34). It is therefore presumable that in the nutrient-deprived TME, where there is also a higher production of TGF-β, mTOR may be inhibited, thereby limiting NK cell effector functions.

## Amino Acid Depletion

In addition to glucose, amino acids are also an important fuel for many cellular processes. Tumors show increased amino acid consumption (74) and synergize with tumor-associated cells to create a nutrient-depleted microenvironment. It has been reported that low arginine concentration impairs the proliferation and IFNγ production of the NK-92 cell line and primary human NK cells (75, 76). In addition, mTOR signaling has been found to be inhibited in leucine-depleted media (27, 28). As previously mentioned, mTOR plays a key role in the modulation of the glycolytic pathway, so the impairment of its signaling cascade may lead to diminished effector functions. Similarly, arginine and glutamine levels also affect mTOR signaling (77). Additionally, mTOR has been found to sustain the initial expression of cMyc, a transcription factor that supports the metabolic reprogramming (including the elevated glycolytic rates) required for the functional responses of IL-2/12-stimulated mouse NK cells (27). Moreover, glutamine and amino acid transport through the SLC7A5 transporter are also required for a sustained expression of cMyc (27).

Our current knowledge indicates that amino acid availability may be necessary for NK cell functionality, although the main role of some amino acids could be to maintain the signaling of other metabolic regulators, such as mTOR or cMyc, rather than to be used as a fuel. Indeed, Loftus et al. have reported that glutaminolysis can be inhibited without reducing NK cell functional responses (27). In this line, a previous report indicated that receptor-induced IFNγ production of murine NK cells was not impaired by limiting concentrations of glutamine (24). These findings suggest that targeting amino acid metabolism may enhance NK cell-based therapies, by impairing tumor fuel supply without reducing NK cells' functionality. However, it is necessary to thoroughly explore metabolic requirements of NK cells to validate this hypothesis and to design better therapeutic strategies.

Nonetheless, it should be also considered that amino acid consumption by tumor and tumor-associated cells leads to the accumulation of immunosuppressive catabolites in the TME. Myeloid-derived suppressor cells (MDSCs) upregulate arginase and inducible nitric oxide synthase (iNOS). Both enzymes use arginine as substrate, and the latter catabolizes its conversion to nitric oxide (NO) (6, 78). It has been found that NO impairs NK cell antibody-dependent cellular cytotoxicity, and that the inhibition of iNOS in a mouse model of breast cancer can rescue this function (79). Furthermore, tumor and tumor-associated dendritic cells and fibroblasts show increased expression of IDO, which catabolizes the conversion of tryptophan to L-kynurenine (80, 81). L-kynurenine can be transported through the SLC7A5 transporter (82), and has been found to inhibit NK cell proliferation (81, 83). Also, L-kynurenine inhibits IL-2-induced upregulation of NKp46 and NKG2D receptors and cytokine production of human NK cells *in vitro* (84).

## Hypoxia

Most solid tumors show high oxygen consumption and disorganized vascularization, which leads to the generation of regions permanently or transiently subjected to hypoxia (85). Cells adapt to this hypoxic conditions through the hypoxia-inducible family of transcription factors (HIFs) that modulate a wide range of genes (86). In particular, most dysregulated genes of NK cells under hypoxia are related to metabolic and biosynthetic processes (87), which is consistent with the results obtained in other cells in which HIF-1α promotes or sustains glycolytic metabolism (88–91). In human NK cells, IL-15-priming synergistically acts with short term hypoxia to induce the upregulation of genes involved in the glycolytic pathway (20). Given the relevance of glycolysis for NK cell effector functions, these findings could suggest that these cells may partially conserve their functionality in hypoxic environments. Indeed, available data argue that hypoxia limits, but does not completely block, NK cell responses (92, 93). However, it is still unclear whether HIF-1α and its effect on glycolytic activity play a significant role on NK cells effector functions. It has been reported that *Hif1a*^−/−^ NK cells have normal metabolic and effector functions in response to IL-2/12 stimulation (27). It would be interesting to study the relationship between HIF-1α, metabolism and effector responses of NK cells under hypoxia. Noteworthy, under hypoxic conditions, there is also a modulation of genes related to immunomodulatory functions and a downregulation of activating receptors, such as NKp30, NKp46, or NKG2D, that contributes to the diminished effector functions (87, 94). Also, hypoxia promotes tumor immune evasion through other mechanisms, such as degrading NK cell-derived granzyme B by autophagy (95). Interestingly, it has been reported that IL-2-priming can increase the expression of the above mentioned activating receptors and improve NK cell cytotoxicity, thereby overcoming the inhibitory effects of hypoxia (93, 96). Considering that there are several antitumor therapeutic strategies based on NK cell stimulation with different cytokines, including IL-2, IL-12, IL-15, IL-18, and IL-21 (29, 31, 32, 97), it would be of great interest to further analyze the effector functions of these cytokine-primed and expanded NK cells under hypoxic conditions.

## Other Factors Influencing NK Cell Metabolism

As before mentioned, NK cell metabolism can be modulated by multiple factors, and some of them may have a relevant impact on the anti-cancer therapeutic efficacy. A recent report revealed that obesity impaired mTORC1 activation and limits NK cell antitumor responses (12). The same article has shown that NK cells from obese individuals have a reduced metabolic response following cytokine stimulation (12). Thus, therapies based in the administration of interleukins may show reduced efficacy in these patients. In contrast, a previous report indicated that NK cells from obese children displayed higher levels of glycolysis and mTORC1 activation both at basal levels and upon cytokine stimulation (13). These data suggest that NK cell metabolism may be differentially regulated in adults and children. It would be also interesting to explore NK cell metabolism in other pathologies, such as viral infection, and test whether the metabolic reprogramming is maintained after a prolonged period. Chronic infections have been related to the exhaustion of effector lymphocytes, and exhausted T cells show a different metabolic pattern (98, 99). It has been described that NK cells can also become exhausted during tumor progression or chronic infections (100, 101). Therefore, it would be of great interest to study metabolic requirements of exhausted NK cells, and furthermore, test whether metabolic reprogramming induced by viral infections and tumors could drive NK cell exhaustion.

## Concluding Remarks

NK cell effector functions are supported by their metabolism. For instance, IFNγ production could be metabolically regulated at different levels, during transcription, translation, or post-translational processing (54). In the restrictive TME, NK cell metabolism and antitumor responses are impaired (38, 58). Similarly, it has been found that tumor cells compete with T cells for glucose, and this metabolic competition in the TME has been described as a driver of cancer progression (57). In accordance, others have demonstrated that highly glycolytic tumors impair T cell antitumor responses through multiple mechanisms (102). Hence, considering that glucose is a key fuel for NK cells, it is presumable that the metabolic competition of the TME will dampen their effector functions. In addition to glucose, reduced amino acid availability and the accumulation of tumor-derived metabolites may also have a substantial impact on NK cell functionality. Thus, targeting tumor metabolism could be a good option to improve the efficacy of NK cell-based therapies. Alternatively, or additionally, NK cell metabolism may be modified to compete more efficiently for nutrients in the TME, or to be less susceptible to the hypoxia-driven inhibition. For instance, stimulation with different cytokine combinations can upregulate the expression of several nutrient transporters, glycolysis and OXPHOS (11, 33, 103), which can be a good option to enhance NK cell metabolic competitiveness, fitness and plasticity, and thus improve their effector functions in the TME. NK cell stimulation with IL-2, IL-15, or IL-18 has been found to increase the expression of amino acid transporters (28, 72). IL-15-priming sustains murine NK cells production of IFNγ in glutamine-free media, and during the inhibition of fatty acid oxidation with etomoxir (24). Similarly, pretreatment with ALT-803, an IL-15 superagonist, confers some metabolic resistance to the inhibition of glycolysis or mTORC1 with 2-deoxy-D-glucose or rapamycin, respectively (25). However, a continuous exposure to IL-15 can lead to NK cell exhaustion, which is accompanied by a reduction in mitochondrial activity (104). It is therefore necessary to continue exploring NK cell metabolism to understand how it could be modified to resist the metabolically restrictive TME and preserve the effector functions. Undoubtedly, immunometabolism is a fascinating field of research that has been demonstrated to be relevant for NK cell effector functions. Further studies will reveal more precisely how TME shapes NK cell metabolism, which represents an attractive target to focus on to improve NK cell-based immunotherapies.

## Author Contributions

All authors listed have made a substantial, direct and intellectual contribution to the work, and approved it for publication.

### Conflict of Interest

The authors declare that the research was conducted in the absence of any commercial or financial relationships that could be construed as a potential conflict of interest.
